# De Novo Transcriptome Assembly Reveals Insights Into Osmoregulation and Oxidative Stress Response in the Gills of the Southern King Crab (*Lithodes santolla*)

**DOI:** 10.1002/ece3.72390

**Published:** 2025-11-05

**Authors:** Alexandra Brante, Paulina Bustos, Claudio Ortega‐Muñoz, Eliana Paola Acuña Gómez, Vicenzo Brante, Rodolfo Farlora

**Affiliations:** ^1^ Programa de Magíster en Ciencias Biológicas mención Biodiversidad y Conservación, Facultad de Ciencias, Instituto de Biología Universidad de Valparaíso Valparaíso Chile; ^2^ Centro de Estudios del Cuaternario de Fuego—Patagonia y Antártica (CEQUA) Punta Arenas Chile; ^3^ Laboratorio de Biotecnología Acuática y Genómica Reproductiva (LABYGER), Facultad de Ciencias, Instituto de Biología Universidad de Valparaíso Valparaíso Chile; ^4^ Laboratorio de Microbiología Integrativa e Innovación Tecnológica (MIIB‐Lab), Facultad de Ciencias, Instituto de Biología Universidad de Valparaíso Valparaíso Chile; ^5^ Centro de Investigación y Gestión de Recursos Naturales (CIGREN) Universidad de Valparaíso Valparaíso Chile

**Keywords:** gill transcriptome, *Lithodes santolla*, osmoregulation, oxidative stress, WGCNA

## Abstract

Understanding the molecular mechanisms underlying physiological adaptations in marine species is crucial for assessing their resilience to environmental stressors. The Southern king crab (*Lithodes santolla*), an ecologically and commercially important species in sub‐Antarctic waters, inhabits dynamic fjord ecosystems characterized by fluctuating salinity, temperatures, and oxygen levels. However, the molecular basis of its adaptive responses remains largely unexplored. In this study, we assembled the first de novo transcriptome of *L. santolla* and compared gene expression between two localities in the Strait of Magellan (Ballena Sound and Choiseul Bay). Sequencing yielded 731,879,912 clean reads, which were assembled into 210,093 transcripts, of which 56,064 contigs were successfully annotated. Differential expression analysis identified 4474 differentially expressed genes (DEGs), with individuals from Ballena Sound exhibiting upregulation of *heat shock proteins* (*HSPs*) and *Na* ^*+*^ */K* ^*+*^ *‐ATPase* (*NKA*), consistent with proteostasis support and ion‐transport demands. Conversely, individuals from Choiseul Bay showed higher expression of *Na* ^*+*^ */H* ^*+*^ *exchanger* (*NHE*), *V‐type proton ATPase* (*V‐ATPase*), and antioxidant enzymes, indicating enhanced pH/ion regulation and redox buffering. Gene Ontology and KEGG enrichment analyses further highlighted ion transport, oxidative stress mitigation, and energy metabolism. Weighted gene co‐expression analysis (WGCNA) recovered modules associated with locality and allowed the identification of high‐connectivity hub genes as candidate biomarkers, yielding a panel for environmental stress monitoring. Taken together, these results reveal the gill‐level coregulation of ion/osmotic regulation, proteostasis, and redox control, enhancing our understanding of *L. santolla's* physiological plasticity. This transcriptomic resource and biomarker candidates provide a valuable molecular framework for hypothesis‐driven experiments and for monitoring the resilience and conservation status of sub‐Antarctic crustaceans under ongoing environmental change.

## Graphical abstract

The Southern King Crab (*Lithodes santolla*), an ecologically and commercially important species in sub‐Antarctic waters. In this study, we assembled the first gill de novo transcriptome of *L. santolla* to characterize gene expression related to environmental responses. Gene Ontology and KEGG enrichment analyses further highlighted key pathways involved in ion transport, oxidative stress mitigation, and energy metabolism. This study enhances our understanding of *L. santolla's* physiological plasticity and provides a valuable molecular framework for future research on environmental resilience and conservation of sub‐Antarctic marine species.

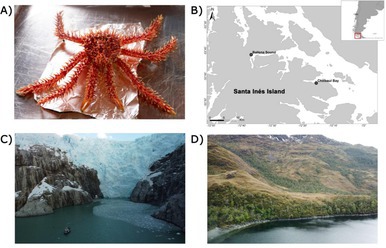

## 1 Introduction

Gills are key organs in marine crustaceans, acting as a multifunctional interface with the environment (Lucu and Towle 2003; Henry et al. 2012). In addition to their primary role in controlling respiratory gas exchange, gills are central to osmoregulation, ion transport, acid–base balance, and immune response (Henry et al. 2012; Thabet et al. 2017). These functions are critical for maintaining homeostasis under environmental fluctuations (McNamara and Faria 2012). Transcriptomic studies in decapods have highlighted the gill's role in responding to environmental stressors, with key genes regulating osmotic balance, oxidative stress, and metabolic processes (Fehsenfeld et al. 2011; Lv et al. 2013; Zhang et al. 2020; Mo et al. 2024; Luo et al. 2024). Despite the expansion of transcriptomic resources in crustaceans, sub‐Antarctic crustaceans remain underrepresented in many areas (Strang and Bosker 2024). At the transcriptomic level, pelagic crustaceans are comparatively well covered, where Antarctic krill ( *Euphausia superba* ) has dedicated resources with an expression atlas across public RNA‐Seq datasets (Sales et al. 2017; Urso et al. 2022), and dominant copepods have recent RNA‐seq studies resolving their environmental and starvation responses (Berger et al. 2024, 2025). However, RNA‐Seq resources for sub‐Antarctic benthic decapods remain, to the best of our knowledge, non‐existent, and available datasets concern a Northern Hemisphere congener, the red king crab ( *Paralithodes camtschaticus* ), reporting analysis in non‐gill tissues (Andersen et al. 2022). Gill‐resolved datasets relevant to ion transport, acid–base regulation, and oxidative balance are non‐existent for sub‐Antarctic lithodids. This knowledge gap limits our understanding of unique physiological adaptations to specific environmental pressures (Pérez‐Moreno et al. 2023).

The Southern king crab (*Lithodes santolla*) is one of the most important artisanal fishery resources in the Magallanes and the Chilean Antarctic Region (Arcos‐Ortega et al. 2019; Molinet et al. 2020). It is also a generalist predator and scavenger, contributing to energy flow and nutrient cycling through trophic interactions (Andrade et al. 2022). It inhabits cold‐temperate and highly variable waters of fjords, channels, and sandy bottoms, particularly in the Strait of Magellan, a unique and highly dynamic environment shaped by glacial valleys inundated by seawater (Arcos‐Ortega et al. 2019; Andrade et al. 2022). These habitats are characterized by pronounced fluctuations in salinity, low temperatures, and seasonal variations in oxygen availability, making them challenging environments that require considerable physiological adaptability (Sepúlveda et al. 2011; Silva and Vargas 2014; Bianchi et al. 2020). Physiologically, *L. santolla* tolerates cold‐temperate environments and is able to tolerate aerial exposure for several days (Urbina et al. 2013; Pretterebner et al. 2019). In Magellanic fjords, it routinely encounters brackish‐to‐marine surface layers, with juvenile occurrence tending toward more marine surface waters (Canete et al. 2017). However, to the best of our knowledge, optimal salinity ranges for this species have not been experimentally defined. Despite its ecological and economic relevance in the Southwest Atlantic and Southeast Pacific oceans, the molecular mechanisms underlying *L. santolla's* ability to cope with environmental variability remain unexplored.

Gills are often the first line of contact with environmental fluctuations (Zhang et al. 2024; Ma and Wang 2024), making them hotspots for studying stress‐related adaptation mechanisms that enable *L. santolla* to cope with environmental fluctuations. Previous transcriptomic analyses in decapods have revealed key genes and pathways involved in ion transport, oxidative stress mitigation, and energy metabolism, underscoring their importance as multifunctional organs in adaptive responses (Fehsenfeld et al. 2011; Lv et al. 2013). However, there is a lack of equivalent data for *L. santolla*, resulting in a substantial deficit in understanding how this sub‐Antarctic species adapts to its habitat's unique and variable conditions.

To address this knowledge gap, we performed the first transcriptomic assembly of *L. santolla*, comparing gill gene expression profiles between two populations from the Strait of Magellan in Southern Patagonia. Through this analysis, we aim to identify differentially expressed genes and enriched biological pathways involved in osmoregulation and environmental stress response. By comparing transcriptomic profiles between populations, we aim to uncover molecular mechanisms that facilitate adaptation to salinity fluctuations, providing insights into the species' physiological plasticity. Understanding these responses is crucial for assessing the resilience of *L. santolla* under changing environmental conditions and contributes to broader conservation efforts for sub‐Antarctic marine species.

## 2 Materials and Methods

### 2.1 Sample Collection, RNA Extraction, and Sequencing

Sampling was conducted in March 2024 at Ballena Sound (53°40′30″ S, 72°37′39″ W) and Choiseul Bay (53°45′34″ S, 72°16′05″ W). Environmental parameters (salinity, temperature, pH, dissolved oxygen, and oxygen saturation) were recorded continuously 12–14 h prior to sampling with a multiparameter probe (WiMo, nke Instrumentation) at 2‐s intervals. To estimate environmental conditions during sampling events, measurements from the final 5‐min period were analyzed, consistent with sampling. Ten individuals of *Lithodes santolla* (*n* = 5 per location) were collected, sexed, and measured for carapace length (CL), carapace width (CW), and weighed (Table S1). A portion of each individual's second posterior left gill pair (counting from the ventralmost pair upward) was dissected and immediately preserved in RNALater solution (Thermo Fisher Scientific), and transported to the laboratory, where they were stored at −80°C. Total RNA was extracted from the posterior gill sample using TRIzol Reagent (Thermo Fisher Scientific), following the manufacturer's protocol. The quantity of total RNA was determined using a NanoDrop Lite (Thermo Fisher Scientific), and its quality was assessed using a Qubit RNA IQ Assay Kit in a Qubit 4 Fluorometer (Thermo Fisher Scientific). Subsequently, total RNA was freeze‐dried and shipped to the sequencing laboratory (Novogene, Sacramento, USA). According to the sequencing protocol, 10 cDNA libraries were prepared at Novogene, using ABclonal Fast RNA‐seq Lib Prep Kit V2 kit and sequenced on Illumina NovaSeq6000 using a paired‐end (2 × 150 bp). The original sequencing files have been uploaded to NCBI's Sequence Read Archive (SRA) under the BioProject ID PRJNA1254097. All procedures and protocols employed in this study were ethically reviewed and approved by the Comité de Ética, Bioética y Bioseguridad of the Universidad de Concepción (approval number CEBB 1081–2021). Field study permissions were granted by the Subsecretaría de Pesca y Acuicultura (E2021‐531 and E‐3315 permits).

### 2.2 De Novo Transcriptome Assembly

Firstly, initial quality control using the FastQC tool (https://www.bioinformatics.babraham.ac.uk/projects/fastqc) was performed for each library. Low‐quality adapters and sequences were filtered using Trimmomatic (v0.39) (Bolger et al. 2014) based on the following settings: ILLUMINACLIP:adapters/TruSeq3‐PE‐2.fa:2:30:10 LEADING:5 TRAILING:5 SLIDINGWINDOW:4:20 MINLEN:150. Trimmed reads were re‐imported to FastQC to check the overall quality of trimming. rRNA reads were sorted and removed by SortMeRNA (v4.3.6) (Kopylova et al. 2012) software using SILVA and Rfam ribosomal databases. Given the unavailability of a reference genome for *Lithodes santolla*, a de novo transcriptome assembly was conducted using Trinity (v2.15.1) (Grabherr et al. 2011) with a minimum contig length of 150 bp.

### 2.3 Assembly Quality and Completeness

The mapping alignment rate was assessed on Bowtie2 (v 2.5.4) (Langmead and Salzberg 2012) mapping the reads back to the assembly. Redundant transcripts were reduced using CD‐HIT (v4.8.1) (Huang et al. 2010): *cd‐hit‐est ‐c 0.95 ‐n 10 ‐M 60000 ‐T 10*. Transcriptome completeness was assessed using Benchmarking Universal Single‐Copy Orthologs (BUSCO, v5.8.2) (Simão et al. 2015) using Arthropoda BUSCO datasets. Assembly statistics were computed before and after removing redundancies using the *TrinityStats.pl* script on Trinity.

### 2.4 Transcriptome Functional Annotation

Transcriptome annotation was performed using Trinotate (v4.0.2) (Bryant et al. 2017). Open reading frames (ORFs) were predicted using TransDecoder (v5.7.1, https://github.com/TransDecoder/TransDecoder), with a minimum length of 200 AA. Sequence similarity searches were conducted with blastp in DIAMOND (v2.1.8) (Buchfink et al. 2021) against the non‐redundant protein NCBI database (nr) (downloaded on November 2, 2024) using the *more‐sensitive* parameter and an E‐value cutoff of 1E‐5. Likewise, conserved protein domain identification was performed using Pfam (v37.2) (Mistry et al. 2021). Functional annotation and ortholog‐based classification, including the assignment of Gene Ontology (GO) terms, KEGG pathways, and COG categories, were performed using the command‐line version of eggNOG‐mapper v2 (Cantalapiedra et al. 2021). Signal peptides identification and their cleavage site locations in proteins from Eukarya were assessed using SignalP (v6.0) (Teufel et al. 2022), while predicted transmembrane regions were predicted using DeepTMHMM (v1.0) (Hallgren et al. 2022). The transcriptome assembly was annotated using *Trinotate_get_feature_name_encoding_attributes.pl* within the Trinity package.

### 2.5 Differential Expression and Enrichment Analyses

Transcription expression was quantified using the mapped‐based method by the Salmon tool (Patro et al. 2017) in the Trinity package with *align_and_estimate_abundance.pl* script. Quasi‐map indices files were merged into raw counts and normalized matrices using the trimmed mean of M values (TMM) method using *abundance_estimates_to_matrix.pl* script within the Trinity package. To assess intra‐ and inter‐site expression similarity prior to differential expression, we computed Pearson correlations among samples based on raw counts with *PtR.pl* script in Trinity package, which supported the PCA and hierarchical clustering structure. Further, differential expression (DE) analysis between the two conditions was analyzed using Bioconductor package DESeq2 (Love et al. 2014) for statistical analysis of the pairwise comparison between conditions, applying the following parameters: a false discovery rate (FDR) ≤ 0.05 and fourfold change to minimize false positives. Before downstream analyses, we only included transcripts with a *p*_adj_ value < 0.05 and |log2FC| ≥ 4 and excluded those differentially expressed transcripts where expression was detected in only one sample. In addition, a hierarchical clustering of features was performed for the dataset, and a heat map was constructed to plot significant differences in gene expression.

Annotated Gene Ontology terms (GO) were classified into Biological Process (BP), Molecular Function (MF), and Cellular Component (CC) categories, with a minimum sequence count of 40 and an e‐value cutoff of 1E‐5. Terms were grouped at level 2 of the GO classification hierarchy to facilitate generalized functional visualization. GO enrichment analysis was then performed using topGO in combination with GO.db and AnnotationDbi packages on R (v4.4.1). Additionally, pathway mapping was performed with the KEGG Automatic Annotation Server (KAAS) and the KEGGREST package on R. KEGG pathway enrichment analysis was carried out using the clusterProfiler package, applying the Benjamini–Hochberg (BH) adjustment method with a significance threshold of *p*_adj_ ≤ 0.05. Venn diagrams were constructed using Venny (v2.1).

### 2.6 Weighted Co‐Expression Network Analysis

Weighted gene co‐expression network analysis (WGCNA) was performed to identify modules associated with between‐site differences and to prioritize hub genes as candidate biomarkers for environmental stress. Starting from the raw count matrix, we retained transcripts with counts ≥ 10 in at least 90% of samples, normalized by TMM, and transformed as log2(TMM + 1). A signed network was built using biweight midcorrelation (bicor) and a soft‐thresholding power of 1 (selected as the smallest power approximating scale‐free topology). Topological overlap (TOM) was computed, and modules were detected via dynamic tree cut (minModuleSize = 30) followed by merging (mergeCutHeight = 0.25). Module–trait relationships were quantified using module eigengenes and the binary factor “Locality” (Ballena vs. Choiseul). Environmental measurements were not modeled as independent continuous traits because they were site‐level and consistently higher in Choiseul, rendering them collinear with locality in this two‐site design. Statistical significance used BH‐adjusted *p*‐values; significant modules were defined as |*r*| ≥ 0.5 with adjusted *p* < 0.05. Hub genes were defined as transcripts with module eigengene‐based connectivity (kME) ≥ 0.70 within significant modules. Module‐level functional enrichment was assessed by over‐representation analysis (ORA) using eggNOG‐mapper annotations for GO and KEGG, with BH‐adjusted *p*‐values and a minimum gene‐set size of 10.

## 3 Results

### 3.1 Environmental Characteristics of Sampling Sites

**FIGURE 1:**
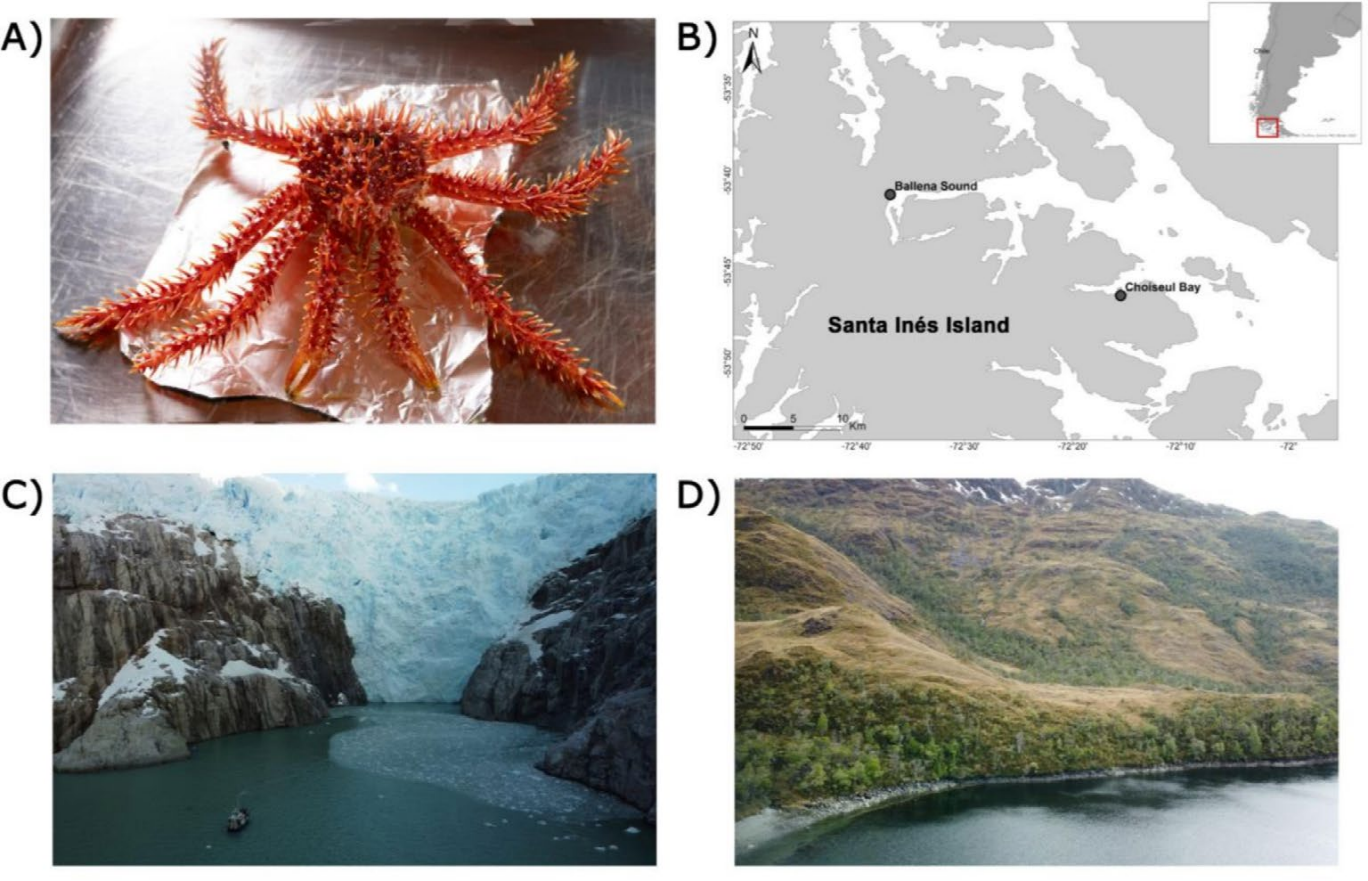
Study organism and contrasting sampling environments in sub‐Antarctic Chilean fjords. (A) The Southern king crab (*Lithodes santolla*). (B) Sampling sites at Ballena Sound and Choiseul Bay are indicated with dots. Both locations are situated on Santa Inés Island. (C) Ballena Sound. (D) Choiseul Bay. Photo credits: (A) Eliana Paola Acuña Gómez, (C‐D) Pedro Valenzuela.

Individuals of *L. santolla* were collected from Ballena Sound and Choiseul Bay in the Strait of Magellan (Punta Arenas, Chile) (Figure 1A). Ballena Sound and Choiseul Bay lie on the north coast of Santa Inés Island and are connected by a narrow channel (Figure 1B). Both systems present topographic constrictions and sills that limit water exchange. Ballena Sound (Figure 1C) shows a fjord‐like configuration influenced by nearby glaciers, with freshwater and fine‐sediment inputs that enhance vertical stratification and yield spatial heterogeneity in chlorophyll and nutrients; intermittent exchanges with the Strait of Magellan occur near the sill. Choiseul Bay (Figure 1D) is comparatively more open but includes local constrictions; exchange with offshore waters is modulated by tides and wind‐driven resuspension, with episodic riverine inputs and photochemical dynamics of dissolved organic matter. Consistent with this setting, in situ measurements indicated marine but distinct water‐mass properties between localities, where Choiseul Bay showed a slightly higher salinity (29.21 PSU vs. 24.66 PSU), temperature (8.74°C vs. 7.63°C), dissolved oxygen (11.17 mg/L vs. 9.86 mg/L), and oxygen saturation (95.36% vs. 92.31%) compared to Ballena Sound (Table S2).

### 3.2 De Novo Gill Transcriptome Assembly and Differential Expression Analysis

A total of 10 cDNA libraries from gills RNA were constructed to create a transcriptome assembly of *L. santolla*. For this, one individual was considered for each library. After quality assessment, trimming, and adapter removal, a total of 731,879,912 clean reads was obtained (Table S3). The final assembly consisted of 210,093 transcripts, of which 155,450 (54.9%) were non‐redundant and subsequently used for downstream analyses (Table 1). The assembly statistics revealed a total length of 177,177,606 bp, with a mean contig length of 1139 bp and an N50 of 1287 bp, indicating a well‐assembled and representative transcriptome. Additionally, the mapping rate of libraries to the reference transcriptome was 79.05%.

**TABLE 1:** Gills de novo transcriptome statistics after redundance removal.

Metrics	De novo transcriptome assembly
Number of transcripts	155,450
Number of Trinity “genes”	116,324
Total bp in assembly	177,177,606
Max contig length	34,226
Min contig length	483
Mean contig length (bp)	1139
Median contig length (bp)	820
%GC	41.41%
N20 contig length	2663
N50 contig length	1287
Number of contigs in N50	38,743
Number of transcripts over 1000 bp	57,963
Mapping rate to reference transcriptome	79.05%
Number of Predicted ORFs	56,064

The completeness of the assembled transcriptome was evaluated using BUSCO analysis with the arthropoda_odb10 dataset. Over the 1013 single‐copy orthologous arthropod genes in OrthoDB, 95.2% of the orthologs were complete, of which 69.6% (705) were single‐copy, and 25.6% (259) were duplicated. Fragmented orthologs accounted for 2.5%, while 2.3% were missing. These results indicate a high‐quality assembly suitable for downstream analysis.

**FIGURE 2:**
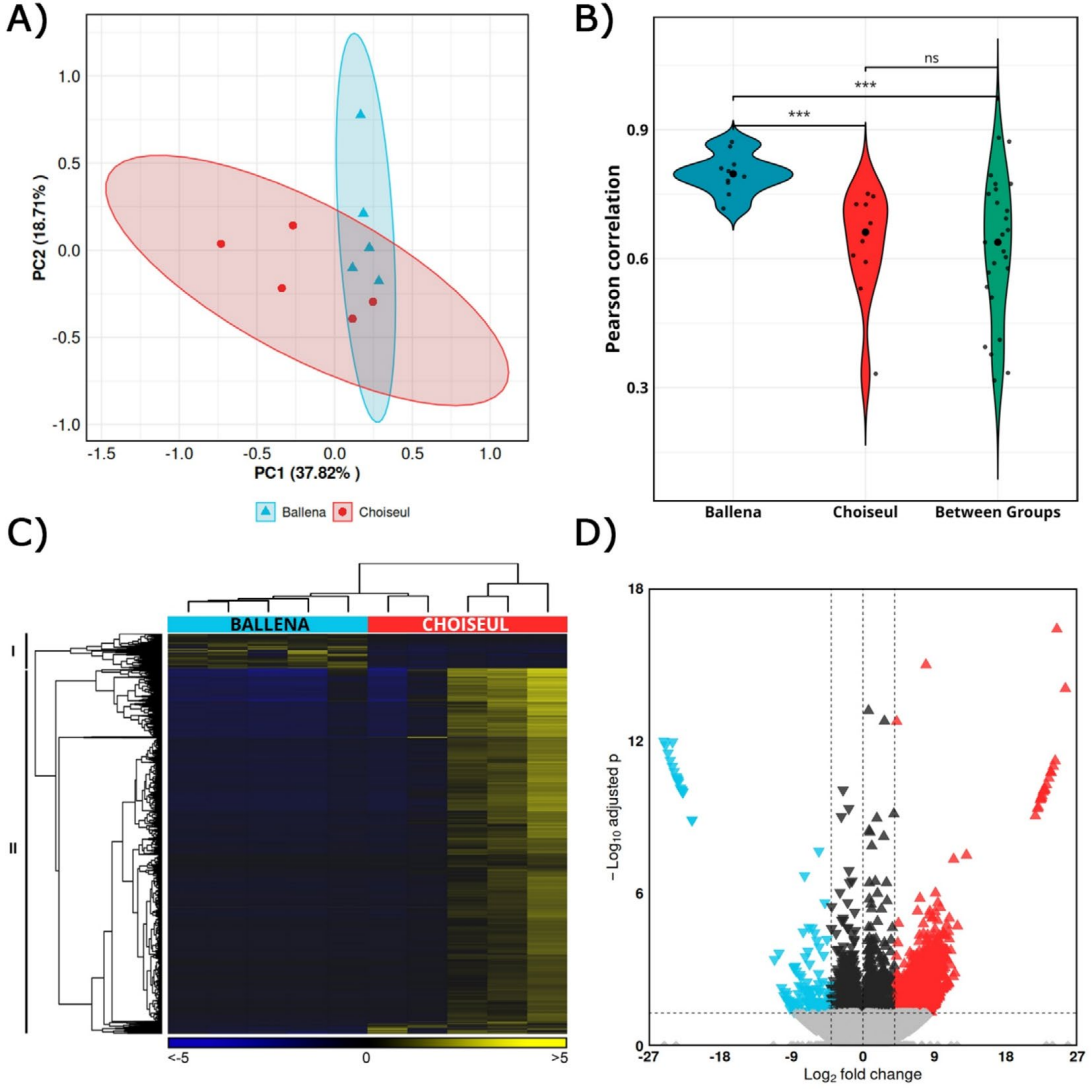
Gill transcriptomic structure and differential expression between Ballena Sound and Choiseul Bay. (A) Principal component analysis (PCA) of gills cDNA libraries from Ballena Sound (blue) and Choiseul Bay (red). Each symbol represents a library. (B) Violin plots of pairwise Pearson correlation coefficients computed within Ballena Sound, within Choiseul Bay, and between localities (dots = individual pairwise values). Pairwise comparisons by Wilcoxon tests: ***p < 0.001; ns, not significant. (C) Hierarchical clustering heatmap of differentially expressed genes (DEGs) from Ballena Sound (blue) and Choiseul Bay (red) samples. DEGs were determined with a minimum |log2 fold change| ≥ 4; false discovery rate (FDR) *p*‐value < 0.05 and values are represented through the color scale from blue (relative low gene expression) to yellow (relative high gene expression). (D) Volcano plot of DEGs between Ballena Sound and Choiseul Bay. Genes upregulated in Ballena are shown in blue, those upregulated in Choiseul in red (|log2 fold change| ≥ 4 vs. FDR < 0.05), and non‐significant transcripts in gray/black.

Principal Component Analysis (PCA) was performed on RNA‐seq data to assess the similarities among samples. The first and second principal components (PC1 and PC2) accounted for 56.53% of the total variance (Figure 2A). The analysis revealed a partial overlapping of two samples between Ballena and Choiseul. To evaluate whether the two deviating Choiseul samples reflected anomalies or natural heterogeneity, we performed a Pearson correlation analysis among samples prior to DE analysis. Within‐group correlations were significantly higher in Ballena compared to Choiseul, whereas between‐group correlations did not differ from those observed in Choiseul (Figure 2B). These findings were consistent with the results of the hierarchical clustering analysis, where these two Choiseul samples were found to cluster with the Ballena group while maintaining proximity to their respective condition (Figure 2C). Overall, 4474 DEGs were identified from pairwise comparisons based on the defined significance thresholds. Of these, 4336 DEGs were uniquely upregulated in Choiseul samples, compared to the remaining 136 DEGs that were upregulated in Ballena samples (Figure 2D, Table S4).

Heat map analysis grouped the differentially expressed genes (DEGs) into two main clusters based on their expression profiles (Figure 2B). The first cluster included transcripts with higher expression in the Ballena condition, whereas the second cluster showed transcripts with higher expression in the Choiseul condition. The Ballena‐associated cluster showed upregulation of genes linked to environmental response, osmoregulation, and innate immunity. DEGs included heat shock proteins such as *HSP20*, *HSP70*, *HSP90*, ion‐transport, and membrane‐communication components such as *Na* ^*+*^ */K* ^*+*^ *‐ATPase*, organic cation transporter, *innexin‐2*, mitochondrial carrier proteins, and immune response factors such as *crustin*, *barrier‐to‐autointegration factor*, *macrophage mannose receptor 1*. Moreover, the Choiseul‐associated cluster showed upregulation of genes linked to osmoregulatory and general stress physiology. Upregulated transcripts were related to heat shock proteins, xenobiotics, and organic anion transport (*ABC‐type organic anion transporter*, *multidrug resistance‐associated protein*), calcium signaling (*voltage‐dependent calcium channels*, *calcium/calmodulin‐dependent protein kinase*), ion exchangers such as *V‐type proton ATPase (ATP6V1/ATP6V0)* and *Na* ^*+*^ */H* ^*+*^ *exchangers*, and antioxidant enzymes (*peroxiredoxin*, *glutathione S‐transferase Mu*, *glutathione peroxidase 4*).

### 3.3 Functional Annotation and Enrichment Analyses

Sequences from de novo transcriptome assembly were annotated using the NCBI NR database. A total of 56,064 (36.0%) contigs showed higher similarity to known proteins in the NR database and therefore were successfully annotated (E‐value cutoff of 1E‐5). Then predicted ORFs were compared to the eggNOG database, retrieving a total of 32,181 (57.4%) mapped annotations.

GO classification revealed general patterns of shared and exclusive terms between Ballena and Choiseul (Figure S1). A total of 16,676 (51.82%) sequences were successfully annotated with GO terms, distributed in 675 unique subcategories within the three main GO categories: Molecular Function (MF), Cellular Component (CC), and Biological Process (BP). Within the MF category, a total of 65 subcategories were shared between the conditions, while 7 subcategories were unique to Ballena and 10 to Choiseul. The CC category included 184 shared subcategories, with Ballena and Choiseul exhibiting 23 and 61 unique terms, respectively. Finally, the category of BP included 270 shared subcategories, with 46 unique to Choiseul and 9 to Ballena. Across all three categories, over 60% of the subcategories were shared between Ballena and Choiseul. The common terms for the CC category were intracellular anatomical structure (1217–2135; 5.49%–9.63%), organelle (1107–1910, 5.00%–8.62%), and cytoplasm (1037–1775, 4.68%–8.00%). The MF category showed a higher representation in terms like protein binding (671–1112, 8.58%–14.21%), organic compound binding (371–622, 4.74%–7.95%), and hydrolase activity (259–522, 3.31%–6.67%). Furthermore, the most represented shared terms at the BP category were regulation of biological process (892–1572, 2.26%–3.99%), regulation of cellular process (838–1459, 2.13%–3.70%), and primary metabolic process (787–1468, 2.00%–3.73%).

**FIGURE 3:**
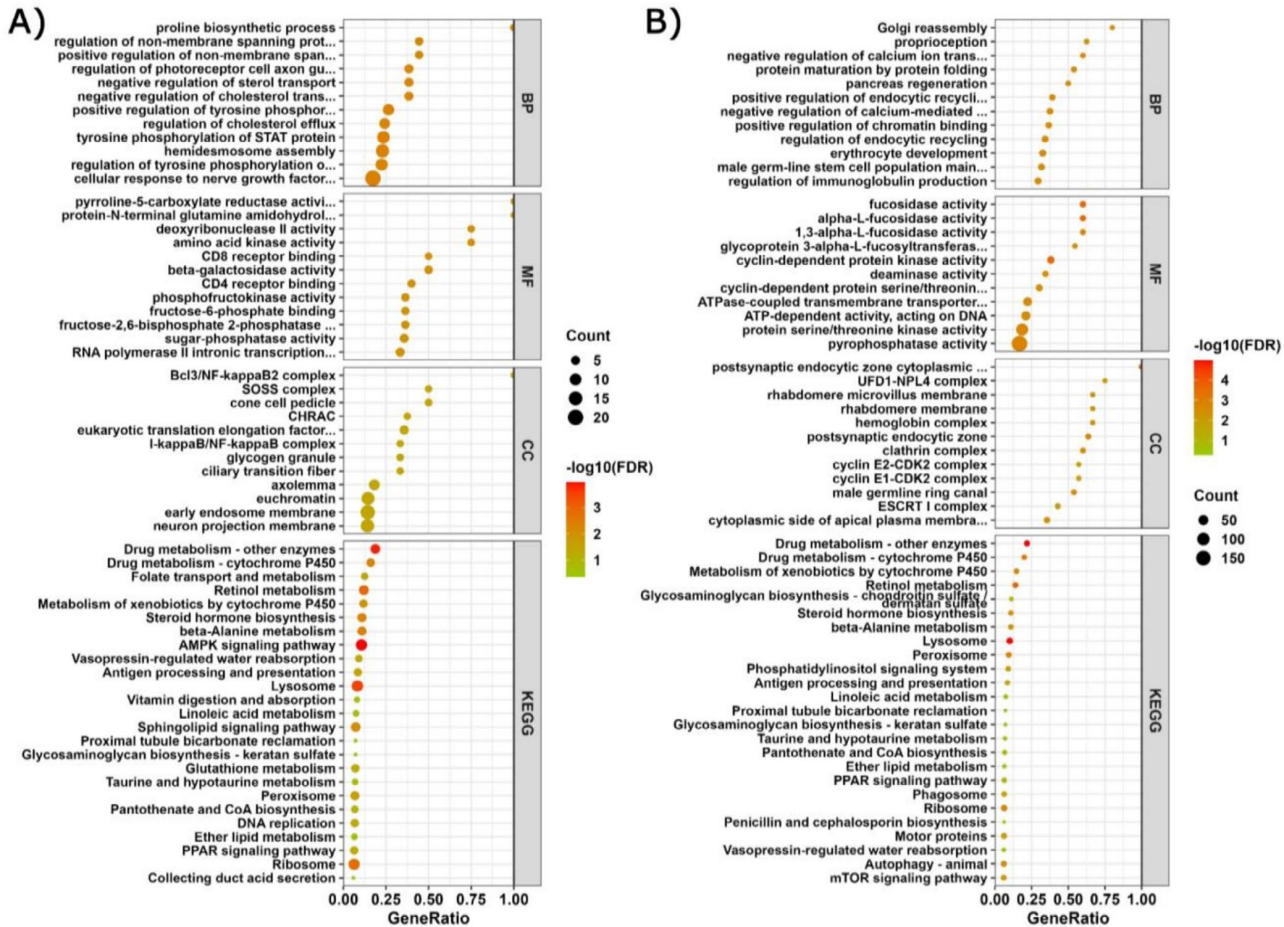
GO term and KEGG pathway enrichment for differentially expressed genes (DEGs) from (A) Ballena Sound and (B) Choiseul Bay. The *x*‐axis represents the gene ratio defined as the proportion of enriched DEGs in GO categories: Molecular Function (MF), Cellular Component (CC), and Biological Process (BP) and KEGG pathways. Dot size represents the number of genes, and the color represents the FDR value.

Functional enrichment analysis was performed considering only DEGs. Notably, GO enrichment analysis revealed no overlapping terms between Choiseul and Ballena among the top enriched terms across all three GO categories (Figure 3, Table S5). For Ballena, upregulated genes were predominantly associated with molecular functions such as CD4/CD8 receptor binding (GO:0042609/GO:0042610) and amino acid kinase activity (GO:0019202). Enriched cellular components included BcI3/NF‐kappaB2 complex (GO:0033257), SOSS complex (GO:0070876), and early endosome membrane (GO:0031901). Within biological processes, the most enriched terms were hemidesmosome assembly (GO:0031581), proline biosynthetic process (GO:0006561), and tyrosine phosphorylation of STAT protein (GO:0007260). In contrast, Choiseul upregulated genes were associated with molecular functions such as alpha‐L‐fucosidase activity (GO:0004560), cyclin‐dependent protein serine/threonine kinase activity (GO:0004693), and deaminase activity (GO:0019239). Enriched cellular components included postsynaptic endocytic zone (GO:0098843), clathrin complex (GO:0071439), and hemoglobin complex (GO:0005833). Likewise, enriched biological processes included negative regulation of calcium‐mediated signaling (GO:0050849), regulation of calcium ion transport into the cytosol (GO:0010523), and asparagine metabolism process (GO:0006528).

Additionally, a total of 14,667 sequences (45.58%) were annotated using the KEGG database. These annotations corresponded to 456 pathways. To further explore the functional implications of the enriched GO terms, KEGG pathways enrichment was performed (Figure 3, Table S6). In Ballena, the enriched pathways included signaling pathways such as AMPK signaling (ko04152) and PI3K‐Akt signaling (ko04151); and response to external stimuli: Vasopressin‐regulated water reabsorption (ko04962), Folate transport and metabolism (ko04981), and Glutathione metabolism (ko00480). On the other hand, Choiseul showed enrichment in signaling pathways such as Phospholipase D signaling (ko04072), Phosphatidylinositol signaling (ko04070), and mTOR signaling (ko04150); and drug metabolism (ko00983).

### 3.4 Module Architecture and Identification of Biomarker Candidates

**FIGURE 4:**
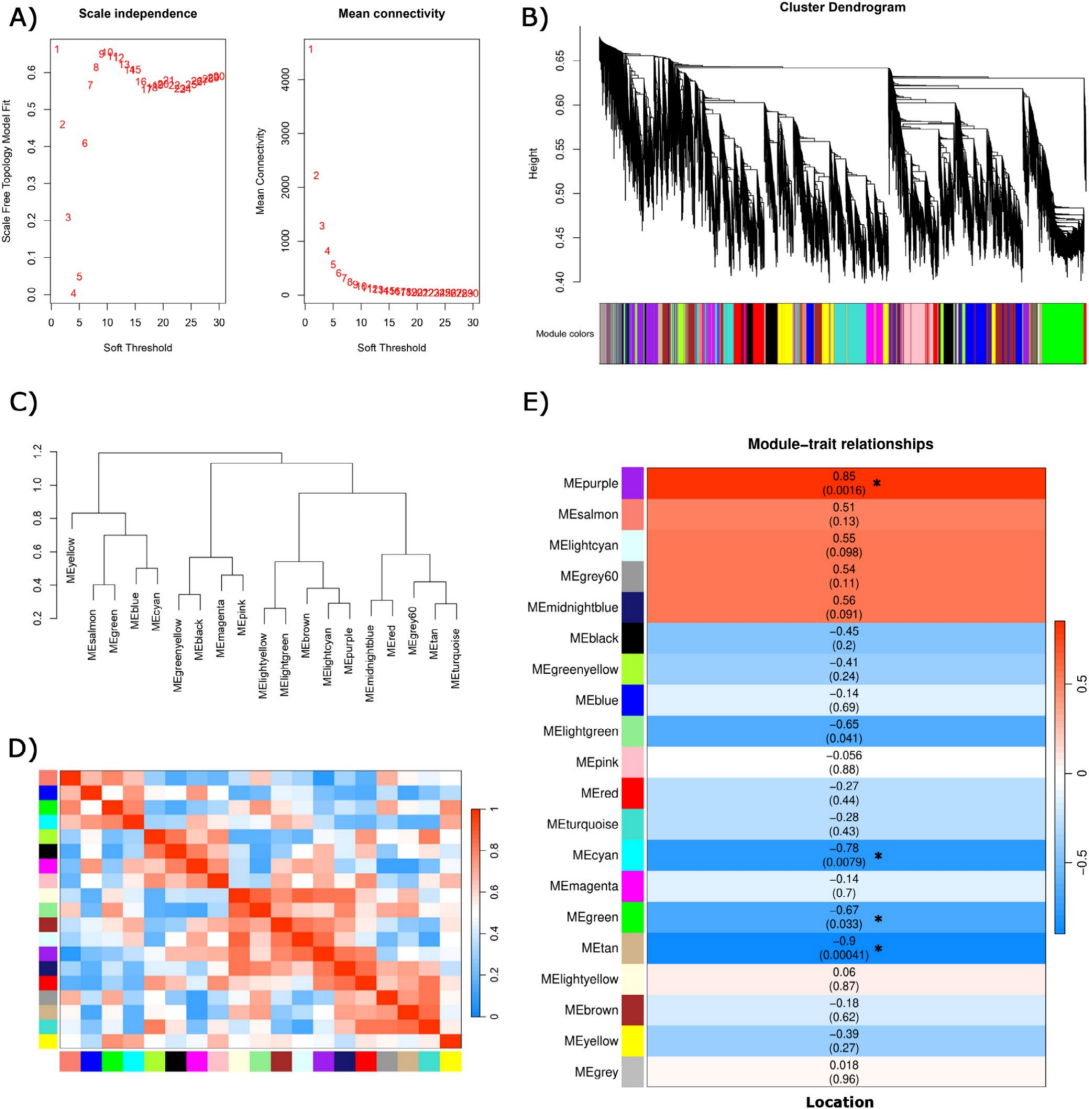
WGCNA of gill transcriptomes and association with locality. (A) Soft‐threshold selection. Scale‐free topology fit index (*R* ^2^, left) and mean connectivity (right) across candidate powers. (B) Gene clustering dendrogram based on TOM dissimilarity with dynamic tree cut; the color bar indicates module assignment. (C) Hierarchical clustering of module eigengenes (MEs). (D) Eigengene correlation (adjacency) heatmap; warm colors denote positive ME–ME correlations and cool colors negative correlations. (E) Module–trait relationships. Heatmap of Pearson correlations between each ME and the binary trait *Location* (Ballena vs. Choiseul). Cells show correlation (*r*) and BH‐adjusted *p*‐value; asterisks mark significance.

After filtering, 13,493 genes from the gill transcriptome were retained and normalized. WGCNA supported a scale‐free topology from a low soft threshold (Figure 4A); we therefore built a signed bicor network using power = 1. Dynamic tree cutting on the TOM identified 20 co‐expression modules (Figure 4B). Module eigengenes clustered into well‐defined clades, and the eigengene adjacency heatmap revealed blocks of positively and negatively correlated modules (Figure 4C,D). Module–trait analysis showed that green, cyan, tan, and purple modules were significantly associated with locality (Ballena vs. Choiseul) (Figure 4E). Within these modules, hub genes defined by kME ≥ 0.7 were purple *n* = 391 (mean 0.808), green *n* = 982 (mean 0.945), cyan *n* = 66 (mean 0.868), and tan *n* = 141 (mean 0.854) (Table S7).

**FIGURE 5:**
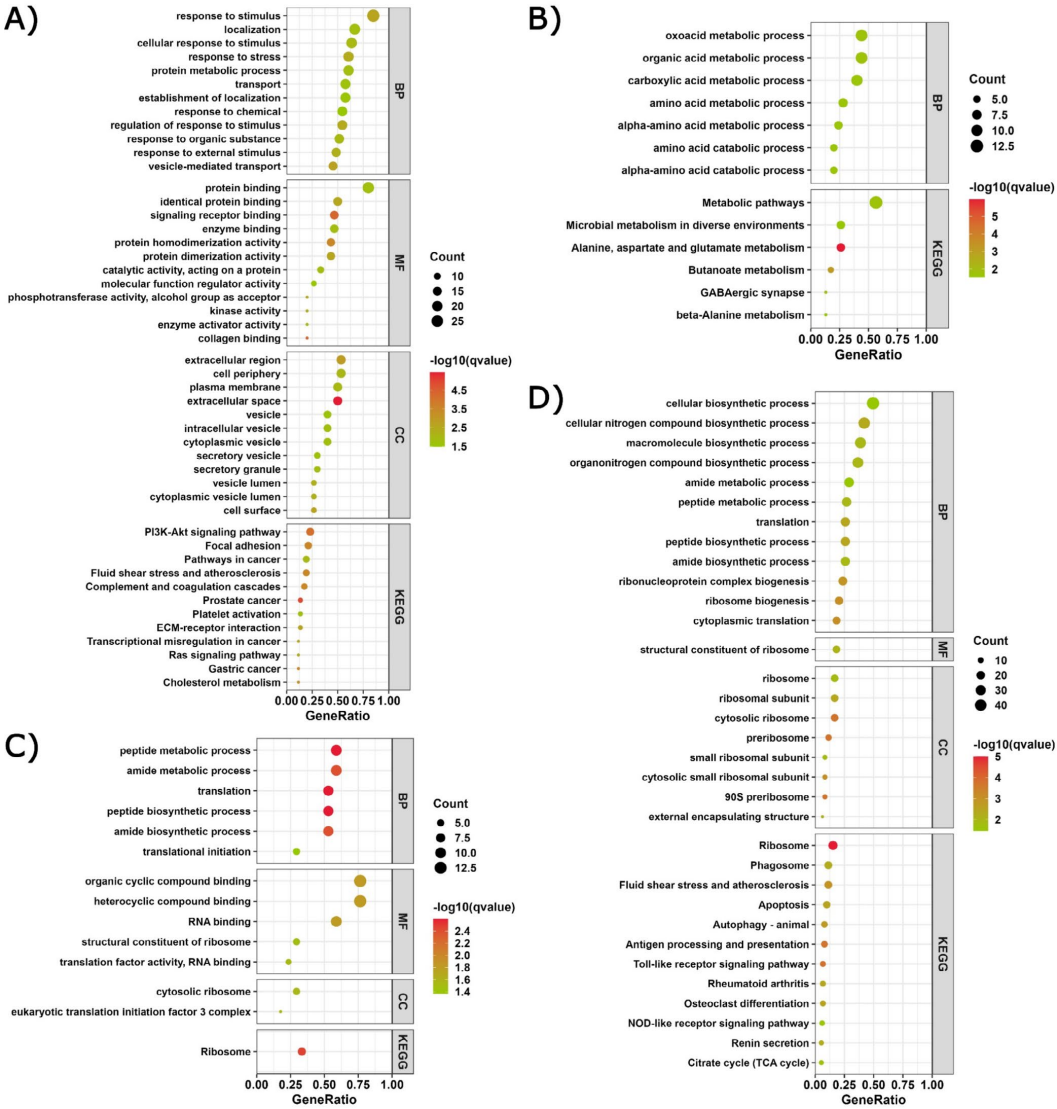
Functional enrichment of locality‐associated WGCNA modules. Bubble plots show over‐represented GO Biological Process (BP), Molecular Function (MF), Cellular Component (CC), and KEGG pathways for (A) purple, (B) tan, (C) cyan, and (D) green modules. The *x*‐axis is the GeneRatio (hits/annotated genes in the module); dot size indicates the number of genes; color encodes −log10(*q*‐value). Only top significant terms are displayed (Benjamini–Hochberg FDR ≤ 0.05).

Functional enrichment of GO terms and KEGG pathways showed different functional profiles in significant modules (Figure 5, Table S8). Cyan/green modules were dominated by translation/proteostasis, including functions related to *ribosome*, *ribosome biogenesis*, and *cytosolic translation*. The tan module included biological and metabolism processes such as *oxoacid metabolic process*, *organic acid metabolic process*, and *carboxylic acid metabolic process*. Whereas the purple module concentrated on ion‐handling/osmoregulation, oxidative/redox, and immune/signaling processes (including r*egulation of calcium‐ion transport* and *positive regulation of reactive oxygen species*). Together, these module‐level signatures and their high‐kME hubs provide network‐based candidate biomarkers responsive to general environmental fluctuations relevant to environmental stress.

## 4 Discussion

Given the ecological and commercial importance of *Lithodes santolla*, understanding its molecular responses to environmental variations is essential for assessing its adaptability and long‐term survival in changing and challenging marine ecosystems (Ramakrishnan 2021; Nielsen et al. 2023). This study presents the first gill transcriptomic profiling of *L. santolla*, providing a high‐quality reference for exploring molecular adaptations (Raghavan et al. 2022). The observed transcriptomic variations between Ballena Sound and Choiseul Bay suggest that habitat heterogeneity influences physiological plasticity (Tanner and Dowd 2019). Because salinity, temperature, and dissolved oxygen co‐varied with locality, we interpret these patterns as associations under natural conditions rather than single‐driver causation, which is consistent with the broader view that multiple environmental factors can modulate acid–base and ion regulatory processes at the gill (Henry et al. 2012). The two localities are hydrologically connected yet shaped by topographic constrictions and sills that restrict water exchange, creating semi‐isolated zones with distinct oceanographic properties. In Ballena Sound, fjord‐like morphology and glacier input freshen, stratify surface waters, and deliver fine particles (Haro et al. 2013). While in Choiseul Bay, a more open setting with tide‐ and wind‐driven resuspension and episodic riverine pulses modulates exchange with offshore waters.

Multivariate ordination showed group separation and revealed greater inter‐individual dispersion in Choiseul relative to Ballena. In line with this, within‐group variances were higher and less variable in Ballena, whereas Choiseul exhibited broader dispersion, and the between‐group distribution more closely resembled Choiseul in terms of both median and variance. The PCA dispersion was predominantly driven by two Choiseul libraries, suggesting within‐site heterogeneity rather than a uniform shift. This pattern is mirrored by a directional asymmetry in the differential expression of the 4474 DEGs, where 4336 were upregulated in Choiseul and only 136 were upregulated in Ballena. Although Choiseul exhibited greater within‐group variance, which generally lowers power to detect differential expression because it depends on effect size, replication, and biological variance, the analysis still revealed a markedly larger set of upregulated transcripts in Choiseul, consistent with a broader transcriptional response at that site (Love et al. 2014; Conesa et al. 2016). In an open‐bay setting such as Choiseul Bay, this heterogeneity may arise from finer‐scale environmental micro‐variation compared with the more semi‐enclosed Ballena Sound, differences in individual physiological state (e.g., molt stage, recent oxygen or osmotic history), and possible demographic mixing if recruitment integrates larvae originating outside the bay, whereas restricted exchange through a narrow channel could promote more locally retained cohorts in Ballena Sound.

Ballena individuals exhibited upregulated expression of genes such as heat shock proteins (*HSPs*) and Na^+^/K^+^‐ATPase (*NKA*). These patterns are common across crustaceans under osmotic or thermal stress, where *HSPs* stabilize proteins and mitigate damage, and are also responsive under hypoxia/reoxygenation (Lucu and Towle 2003; Mengal et al. 2023; Jeyachandran et al. 2023; González‐Ruiz et al. 2023). For *NKA*, euryhaline crabs often show higher branchial activity in diluted seawater. For example, the mud crab (*Scylla paramamosain*) exposure to lower salinity levels increased the *NKA* activity (Xu et al. 2023), which is typical when reduced external salinity forces stronger ion‐uptake activity (Tsai and Lin 2007). Nonetheless, branchial *NKA* supports several physiological processes aside from sodium balance, including a central role in ammonia handling and acid‐balance regulation; its transport activity can also interact directly with ammonium (Tsai and Lin 2007; Henry et al. 2012). Moreover, *HSPs* respond to multiple stressors across decapods and other crustaceans, such as temperature change, hypoxia, pH shifts, metal, and pathogens (Mengal et al. 2023). Likewise, *HSP70* and *HSP90* have been reported to interact with *NKA* under temperature and salinity stress in the whiteleg shrimp ( *Litopenaeus vannamei* ) (Giffard‐Mena et al. 2024). The upregulation of *HSPs* and *NKA* in *L. santolla* may suggest that proteostasis and ion transport are involved in stabilizing osmotic and acid–base status under naturally co‐varying temperatures, oxygen levels, and salinity. However, future studies are needed to validate their roles in *L. santolla* under controlled manipulations of temperature, oxygen, and salinity.

Moreover, Choiseul showed higher expression of transcripts related to osmoregulation and oxidative stress, consistent with the broader transcriptional shift and the greater within‐site dispersion observed at that locality. Upregulated transcripts included key osmoregulatory proteins such as *Na* ^*+*^ */H* ^*+*^ *exchangers* (*NHE*) and *V‐type H* ^*+*^ *‐ATPase*, which participate in ion balance and pH regulation in decapods (McNamara and Faria 2012; Rahi et al. 2018; Lucu and Turner 2024). *NHE* and *V‐ATPase* are crucial for maintaining ion balance and pH regulation in decapods. While roles for *NHE* and *V‐ATPase* are well supported in freshwater crustaceans and have been related to osmoregulation in low‐ionic environments (Rahi et al. 2018), marine adaptations may differ. In marine and intertidal crabs, *V‐ATPase* and *NHE* are also frequently associated with acid–base regulation and ammonia handling (Weihrauch et al. 2009; Zhang et al. 2021). Their involvement in *L. santolla* requires further physiological validation. Additionally, stress chaperones and antioxidant enzymes, such as *peroxiredoxin* (*PRDX*), *glutathione S transferase Mu* (*GST*), and *glutathione peroxidase 4* (*GPx4*), were upregulated. Studies in other crustaceans have shown that glutathione‐based defenses are sensitive to salinity and temperature shifts. This adjustment is due to an increase in metabolic demand and the production of reactive oxygen species (ROS) when the environment changes, resulting in the activation of antioxidant enzymes to maintain redox homeostasis (Frías‐Espericueta et al. 2022). Exposure to variable salinities also modulates antioxidant enzymes in swimming crabs ( *Callinectes danae* and *C. ornatus* ). Specifically, a hypersaline challenge has been demonstrated to elevate the activity of antioxidant enzymes such as *GST* and *GPx* in gills (Freire et al. 2011). Nonetheless, the functional roles of these antioxidant genes in *L. santolla* remain putative, and targeted experiments are necessary to resolve their responses and roles across controlled variables, thereby defining how antioxidant defenses and ion‐transport pathways are coordinated in this species.

Across localities, enrichment analyses revealed a core set of shared energy and redox pathways that have been commonly reported in decapods under controlled temperature, oxygen, and salinity gradients. In decapods exposed to changes in salinity, AMPK and PI3K–Akt were frequently tied to shifts in glycolysis and cellular energy use. For instance, in black tiger shrimp ( *Penaeus monodon* ), salinity stress induced the activation of PI3K–Akt and AMPK pathways (Li, Si, et al. 2023). Likewise, exposure to salinity stress in Oriental river prawn ( *Macrobrachium nipponense* ) led to enrichment of AMPK and PI3K–Akt together with lysosome and drug‐metabolism pathways (Xue et al. 2022; Li, Ye, et al. 2023). Glutathione and xenobiotic/drug‐metabolism enrichment align with other decapod studies where salinity and oxidative stress have been shown to modulate antioxidant enzymes and oxidative‐damage indices, indicating plastic redox buffering (Freire et al. 2011; Frías‐Espericueta et al. 2022; Li, Ye, et al. 2023). These patterns may suggest plastic detoxification mechanisms in varying environments and potentially reflect the need to control ROS and to clear endogenous oxidation products in *L. santolla*, and future studies will be needed to confirm these findings. Moreover, mTOR, phospholipase D, and phosphatidylinositol signaling enrichment support growth‐control regulation and membrane‐signaling modules. mTOR plays a crucial role in regulating molting in crustaceans (Mykles 2021; Hou et al. 2021). In the red king crab ( *Paralithodes camtschaticus* ), expression of components of the mTOR pathway in molt‐related tissues was associated with growth processes and temperature‐sensitive control of the molt cycle (Andersen et al. 2022). Future studies should explore whether this pathway's activation is associated with molting cycles and environmental stress in *L. santolla* individuals.

The WGCNA resolved 20 co‐expression modules, four of which were significantly associated with locality. Particularly, the purple module integrated oxidative‐stress signaling and ion transport at the gill interface, with enrichment for *positive regulation of reactive oxygen species metabolic process* (GO:2000379) and for *regulation/positive regulation of calcium‐ion transport* (GO:0051928). In sub‐Antarctic fjords, meltwater pulses and resuspension of fine sediments can introduce glacier‐derived particulates, dissolved organic matter (DOM), and trace metals into nearshore waters, restructuring coastal biogeochemistry and light/particle fields, promoting ROS formation via photochemical and redox‐cycling pathways (Hawkings et al. 2014; Marshall et al. 2021; Morris et al. 2022). Sustained or repeated ROS excursions can overwhelm antioxidant buffering, elevate lipid peroxidation and protein oxidation, and divert energy from growth and reproduction to maintenance, with consequences for survival if physiological limits are exceeded (Freire et al. 2011; Fanjul‐Moles and Gonsebatt 2011; Frías‐Espericueta et al. 2022). Framed in this context, the upregulation of glutathione‐based enzymes together with *HSPs* and ion‐transport machinery in *L. santolla* is consistent with the gill's integrated roles in proteostasis, redox control, and acid–base/ammonia handling across environments that differ in the source of oxidative pressure (glacially influenced vs. open, resuspension‐driven). WGCNA‐based hub genes enable the identification of biomarker candidates (Sánchez‐Baizán et al. 2022; Alfano et al. 2023). Therefore, hub genes within the purple module may serve as biomarker candidates for environmental stress monitoring.

Overall, our results reinforce the importance of understanding the molecular responses of *L. santolla* to environmental stressors, as these insights might help evaluate the species' ability to withstand environmental changes, identify molecular markers of stress, and support conservation strategies aimed at mitigating potential risks. Climate change‐driven alterations in salinity, temperature, and oxygen availability may impact the species' resilience by challenging its physiological homeostasis (Doney et al. 2012). The observed upregulation of stress‐response pathways, particularly *HSPs*, AMPK signaling, and antioxidant defenses, suggests that *L. santolla* exhibits molecular plasticity that may allow it to adjust to its habitat fluctuations. These markers could be used to assess population health and detect early physiological stress, informing conservation strategies aimed at mitigating environmental pressures. In addition, monitoring these transcriptomic responses through time can help identify thresholds beyond which stress responses become maladaptive, providing insight into whether populations are approaching their physiological limits. Understanding how transcriptomic responses vary across different habitats and stress conditions is essential for predicting the species' resilience to climate change and habitat degradation. The integration of these molecular insights into conservation and resource management may contribute to identifying at‐risk populations and prioritizing conservation actions to ensure the long‐term viability of *L. santolla* in sub‐Antarctic ecosystems.

## Author Contributions

**Alexandra Brante:** data curation (lead), formal analysis (lead), investigation (equal), methodology (lead), visualization (lead), writing – original draft (lead). **Paulina Bustos:** conceptualization (equal), supervision (equal), writing – review and editing (equal). **Claudio Ortega‐Muñoz:** investigation (equal), methodology (supporting). **Eliana Paola Acuña Gómez:** conceptualization (equal), funding acquisition (lead), investigation (equal), writing – review and editing (equal). **Vicenzo Brante:** data curation (supporting), software (lead). **Rodolfo Farlora:** conceptualization (equal), supervision (equal), writing – review and editing (equal).

## Conflicts of Interest

The authors declare no conflicts of interest.

## Supporting information


**Appendix S1:** ece372390‐sup‐0001‐AppendixS1.docx.

**Appendix S2:** ece372390‐sup‐0002‐AppendixS2.xlsx.

## Data Availability

The raw RNA‐Seq datasets used in this study are publicly available at the NCBI Sequence Read Archive under the BioProject PRJNA1254097. Additional processed data is available from the corresponding author upon reasonable request.
