# Supplementary material for: De Novo Transcriptome Assembly Reveals Insights Into Osmoregulation and Oxidative Stress Response in the Gills of the Southern King Crab (Lithodes santolla)
Source: Ecol Evol. 2025 Nov 5;15(11):e72390. doi: 10.1002/ece3.72390 (PMC12588808; doi:10.1002/ece3.72390)
Supplement: Supplementary file 1 — Appendix S1: ece372390‐sup‐0001‐AppendixS1.docx. [file ECE3-15-e72390-s002.docx]

## De Novo Transcriptome Assembly Reveals Insights into Osmoregulation and Oxidative Stress Response in Southern King Crab (*Lithodes santolla*) Gills

Alexandra Brante ^1,2,3,4^, Claudio Ortega-Muñoz ^1,2,3^, Paulina Bustos ^4,5^, Eliana Paola Acuña Gomez ^3^, Vicenzo Brante ^4^ and Rodolfo Farlora ^2,5^ *

*Corresponding author: R. Farlora, Laboratorio de Biotecnología Acuática y Genómica Reproductiva (LABYGER), Facultad de Ciencias, Universidad de Valparaíso, Gran Bretaña 1111, Playa Ancha, Valparaíso 2360102, Chile. Phone Number: +56322508200. E-mail: [rodolfo.farlora@uv.cl](mailto:rodolfo.farlora@uv.cl) ORCID ID: <https://orcid.org/0000-0002-0048-1102>.

1. Programa de Magíster en Ciencias Biológicas mención Biodiversidad y Conservación, Instituto de Biología, Facultad de Ciencias, Universidad de Valparaíso.

2. Laboratorio de Biotecnología Acuática y Genómica Reproductiva (LABYGER), Instituto de Biología, Facultad de Ciencias, Universidad de Valparaíso, Valparaíso, Chile.

3. Centro de Estudios del Cuaternario de Fuego - Patagonia y Antártica (CEQUA), Punta Arenas, Chile

4. Laboratorio de Microbiología Integrativa e Innovación Tecnológica (MIIB-Lab), Instituto de Biología, Facultad de Ciencias, Universidad de Valparaíso, Valparaíso, Chile

5. Centro de Investigación y Gestión de Recursos Naturales (CIGREN), Universidad de Valparaíso, Valparaíso, Chile.

**Supplementary information**


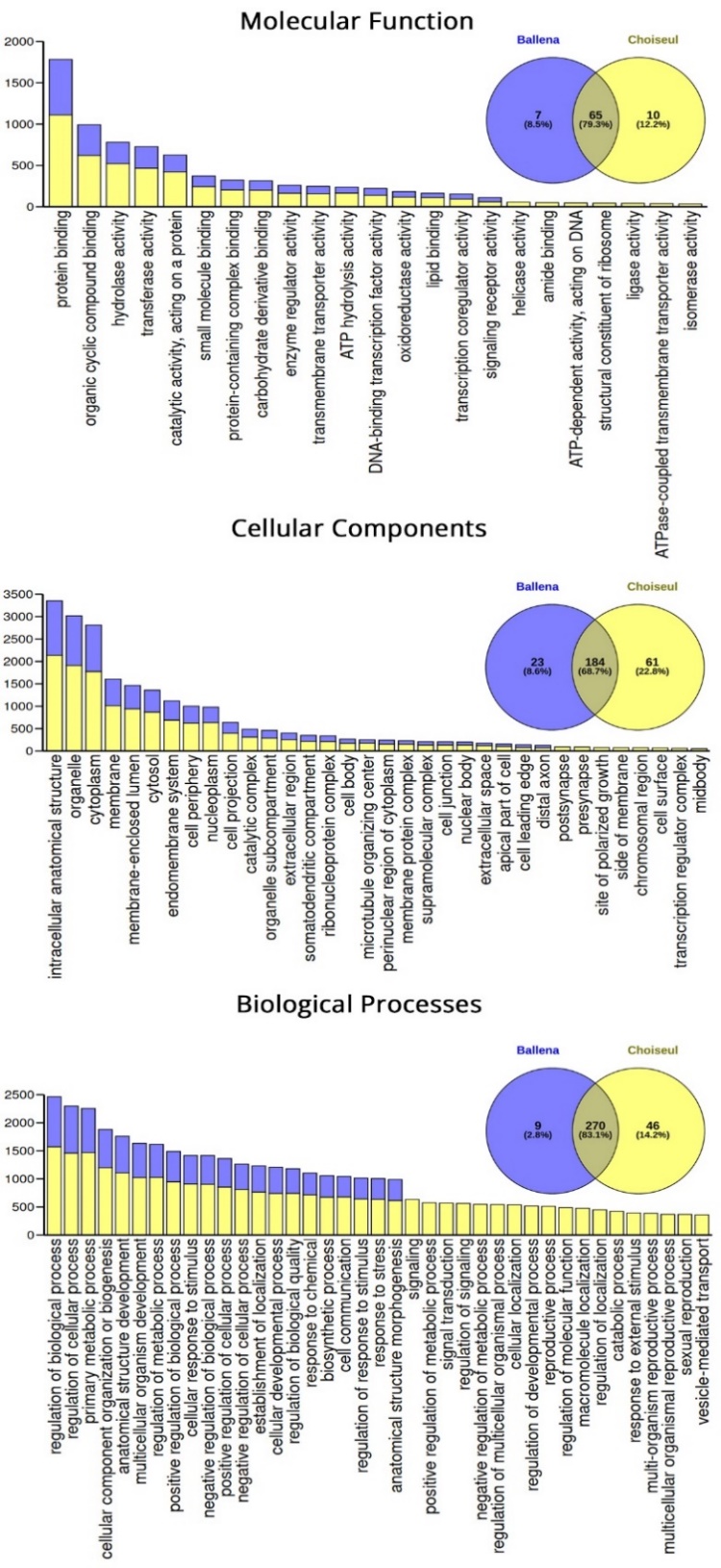


**Figure S1** Distribution of Gene Ontology (GO) terms for the annotated transcripts. The staked bar plot provides the distribution of subcategories of Molecular Function, Cellular Component, and Biological Process categories in Ballena Sound (purple) and Choiseul Bay (yellow) conditions. Venn diagrams show the specific and common GO terms among the three categories, with number and percentage of counts for each category.

**Table S1** Morphometric measurements of individuals collected from Ballena Sound and Choiseul Bay.

| **Sampling site** | **Sample** | **Sex** | **CL (mm)** | **CW (mm)** | **Weight (g)** |
| --- | --- | --- | --- | --- | --- |
| Ballena Sound | 122 | Male | 111.0 | 85.0 | 385 |
|  | 123 | Male | 74.4 | 62.4 | 185 |
|  | 124 | Male | 89.5 | 80.6 | 323 |
|  | 126 | Male | 85.5 | 69.7 | 238 |
|  | 127 | Male | 86.5 | 73.3 | 299 |
| Choiseul Bay | 110 | Male | 99.3 | 80.0 | 248 |
|  | 111 | Male | 91.1 | 75.2 | 227 |
|  | 112 | Male | 88.4 | 68.2 | 212 |
|  | 115 | Male | 75.5 | 62.0 | 138 |
|  | 119 | Male | 66.0 | 55.0 | 96.0 |

**Table S2** Physicochemical differences of sampling sites.

| **Sampling site** | **Date** | ***pH*** | **T (°C)** | **Dissolved Oxygen (mL/L)** | **Oxygen Saturation (%)** | **Salinity (PSU)** |
| --- | --- | --- | --- | --- | --- | --- |
| Ballena Sound | March 2024 | 8.03 | 7.63 | 9.86 | 92.31 | 24.66 |
| Choiseul  Bay | March 2024 | 8.03 | 8.74 | 11.17 | 95.36 | 29.21 |

**Table S3** RNA extraction quality and sequencing read statistics from *Lithodes santolla* samples in Ballena Sound and Choiseul Bay.

| **Sampling site** | **Sample** | **Total RNA (μg)** | **A260/280** | **IQ** | **Raw reads** | **Clean reads** |
| --- | --- | --- | --- | --- | --- | --- |
| Ballena Sound | 122 | 59.5204 | 2.10 | 8.1 | 71,635,008 | 62,555,888 |
|  | 123 | 13.6538 | 2.00 | 9.4 | 91,479,750 | 79,051,520 |
|  | 124 | 16.8562 | 2.05 | 8.0 | 88,331,556 | 78,249,608 |
|  | 126 | 31.8315 | 2.12 | 7.4 | 83,227,590 | 73,151,560 |
|  | 127 | 23.6049 | 2.14 | 7.9 | 84,737,396 | 74,137,898 |
| Choiseul Bay | 110 | 32.2279 | 2.02 | 7.6 | 75,298,306 | 71,625,038 |
|  | 111 | 34.7255 | 2.01 | 7.6 | 83,149,504 | 74,278,018 |
|  | 112 | 22.8317 | 2.11 | 8.1 | 80,134,246 | 71,256,306 |
|  | 115 | 22.6785 | 2.09 | 8.4 | 82,945,658 | 73,109,968 |
|  | 119 | 23.9112 | 2.12 | 7.8 | 86,511,346 | 74,464,108 |
